# Resuspension and atmospheric transport of radionuclides due to wildfires near the Chernobyl Nuclear Power Plant in 2015: An impact assessment

**DOI:** 10.1038/srep26062

**Published:** 2016-05-17

**Authors:** N. Evangeliou, S. Zibtsev, V. Myroniuk, M. Zhurba, T. Hamburger, A. Stohl, Y. Balkanski, R. Paugam, T. A. Mousseau, A. P. Møller, S. I. Kireev

**Affiliations:** 1 Norwegian Institute for Air Research (NILU), Department of Atmospheric and Climate Research (ATMOS), Kjeller, Norway; 2 National University of Life and Environmental Sciences of Ukraine, Kiev, Ukraine; 3 CEA-UVSQ-CNRS UMR 8212, Institut Pierre et Simon Laplace, Laboratoire des Sciences du Climat et de l’Environnement (LSCE), L’Orme des Merisiers, F-91191 Gif-sur-Yvette Cedex, France; 4 King’s College London, London, United Kingdom; 5 Department of Biological Sciences, University of South Carolina, Columbia, SC 29208, USA; 6 Laboratoire d’Ecologie, Systématique et Evolution, CNRS UMR 8079, Université Paris-Sud, Bâtiment 362, F-91405 Orsay Cedex, France; 7 Deputy General Director of the State Enterprise “Chernobyl Special Kombinat”, Chernobyl city, 6 Shkolna street, Ukraine

## Abstract

In April and August 2015, two major fires in the Chernobyl Exclusion Zone (CEZ) caused concerns about the secondary radioactive contamination that might have spread over Europe. The present paper assessed, for the first time, the impact of these fires over Europe. About 10.9 TBq of ^137^Cs, 1.5 TBq of ^90^Sr, 7.8 GBq of ^238^Pu, 6.3 GBq of ^239^Pu, 9.4 GBq of ^240^Pu and 29.7 GBq of ^241^Am were released from both fire events corresponding to a serious event. The more labile elements escaped easier from the CEZ, whereas the larger refractory particles were removed more efficiently from the atmosphere mainly affecting the CEZ and its vicinity. During the spring 2015 fires, about 93% of the labile and 97% of the refractory particles ended in Eastern European countries. Similarly, during the summer 2015 fires, about 75% of the labile and 59% of the refractory radionuclides were exported from the CEZ with the majority depositing in Belarus and Russia. Effective doses were above 1 mSv y^−1^ in the CEZ, but much lower in the rest of Europe contributing an additional dose to the Eastern European population, which is far below a dose from a medical X-ray.

On Sunday 26^th^ April 2015 at 23.30 (local time), exactly 29 years after the Chernobyl Nuclear Power Plant (CNPP) accident, a massive fire started in the Chernobyl Exclusion Zone (CEZ). The next morning (April 27^th^) at 07.30 the fire was partially stabilised and the fire-fighters focused on only two areas of 4.2 and 4.0 hectares. However, the fire spread to neighbouring areas due to the prevailing strong winds. During the night of April 27^th^ to 28^th^, 2015, the fire spread to areas close to the Radioactive Waste Disposal Point (RWDP), and burned around 10% of the grassland area at the western of the RWDP^1^. On April 29^th^ and 30^th^, 2015, the attempts to stop the fires in the CEZ did not succeed. Fire brigades from Chernobyl and Kiev region supported extinguishing attempts and the last 70 ha were suppressed on May 2^nd^, 2015. The radiation background is continuously monitored in the CEZ by an automated radiation monitoring system (ARMS) at 39 points^1^. Given the importance of this fire, background radiation and radionuclide content in the air near the fire were also analysed online.

Another less intensive fire episode took place in August 2015. About 32 hectares were initially burned in the CEZ on August 8^th^ 2. The fires started at three locations in the Ivankovsky area. As of 07.00 on August 9^th^, the fires had been reportedly localized and fire-fighters continued to extinguish the burning of dry grass and forest. The same fire affected another forested area, known as Chernobylskaya Pushcha. The fire spread through several abandoned villages located in the unconditional (mandatory) resettlement zones of the CEZ and ended on August 11^th^.

Forest fires can cause resuspension of radionuclides in contaminated areas^3^. This has caused concern about possible fires in heavily contaminated areas such as the CEZ^4^. While concerns were initially limited to the vicinity of the fires, Wotawa *et al.*^5^ have shown that radionuclides resuspended by forest fires can be transported even over intercontinental distances. Earlier in 2015, Evangeliou *et al.*^6^, based on a detailed analysis of the current state of the radioactive forests in Ukraine and Belarus, reported that forest cover in the CEZ has increased from about 50% in 1986 to more than 70% today. Precipitation has declined and temperature has increased substantially making the ecosystem vulnerable to extensive drought. Analysis of future climate using IPCC’s (Intergovernmental Panel on Climate Change) REMO (REgional MOdel) A1B climatic scenario^7^ showed that the risk of fire in the CEZ is expected to increase further as a result of drought accompanied by lack of forest management (e.g. thinning) and deteriorating fire extinguishing services due to restricted funding. The same group^8^ considered different scenarios of wildfires burning 10%, 50% and 100% of the contaminated forests. They found that the associated releases of radioactivity would be of such a magnitude that it would be identical to an accident with local and wider consequences^9^. The additional expected lifetime mortalities due to all solid cancers could reach at least 100 individuals in the worst-case scenario.

This paper aims at defining the extent of the radioactive contamination after fires that started in the CEZ on April 26^th^ (ended 7 days after) and August 8^th^ (ended 4 days after) 2015. We study the emission of the labile long-lived radionuclides ^137^Cs (t_½_ = 30.2 y) and ^90^Sr (t_½_ = 28.8 y) and the refractory ^238^Pu (t_½_ = 87.7 y), ^239^Pu (t_½_ = 24,100 y), ^240^Pu (t_½_ = 6,563 y) and ^241^Am (t_½_ = 432.2 y). These species constitute the radionuclides remaining in significant amounts since the Chernobyl accident about 30 years ago, and their deposition has been monitored continuously by the Ukrainian authorities. The respective deposition measurements have been adopted from Kashparov *et al.*^10^,^11^ and are stored in NILU’s repository website (http://radio.nilu.no). Using an atmospheric dispersion model, we simulate the atmospheric transport and deposition of the radioactive plume released by the forest fires. We also estimate the internal and external exposure of the population living in the path of the radioactive smoke. We assess the significance of the emissions with respect to the INES scale and define the regions over Europe, which were the most severely affected.

## Results

### Vegetation in the burned area

Table 1 reports land cover types in the areas burned in April and August 2015 from GlobCover 2009 (300 m resolution)^11^ and direct observation of the CEZ prior to the fires. The estimated total area affected by fires in spring 2015 was 10,882 hectares. About 41% of this was former agricultural land left to its fate since 1986 after the mandatory evacuation of the CEZ. Another 41% includes forested lands (artificial and natural forests). Artificial forest includes scots pine trees planted after the accident. They constitute local species of semi-natural forest according to FAO classification^13^. The remaining areas split were mainly swamps that have been excluded from the simulation, as it is unclear to what extent these areas actually burned. This gives a total burned area considered for our radionuclide resuspension calculations of 9,241 hectares.

The burned area was significantly smaller during the fires in August 2015 with a total of 5,867 hectares (see Table 1). About 51% of these were natural forests (populated by seeds or vegetative natural regeneration from previous generation of stands), 26% were artificial forests planted with a tree spacing of 2.5–3.0 m (plantations) and 19% were former agricultural land. The remaining area affected by the summer 2015 fires can be seen in Table 1. Overall, the total active burned area was estimated to be 5,698 hectares.

### Emission and transport of radionuclides

Based on radionuclide measurements in the area^6^,^14^, it is estimated that about 2–9% of ^137^Cs and less than 10% of ^90^Sr of the initial Chernobyl release (85 PBq of ^137^Cs and 10 PBq of ^90^Sr), still remain in the contaminated forests of Ukraine and Belarus. For ^238–240^Pu and ^241^Am, the remaining activity burdens are in the order of a few TBq. Emissions of ^137^Cs, with a total of 810 mg for the spring fires and 2,527 mg for the summer fires, were the largest of all released radionuclides. For comparison, about 26 kg of ^137^Cs were released after the CNPP accident, yielding a resuspended fraction of about 0.1 per mille. Emissions were larger in summer than in spring despite the smaller burned area, because the burned area was more heavily contaminated. Plutonium-239 and 240 were also released in significant amounts with a total of 1250 and 505 mg in spring and 1479 and 598 mg in summer, respectively. Strontium-90 and ^241^Am emissions were even lower, while ^238^Pu was released only in trace amounts.

**Figure 1:**
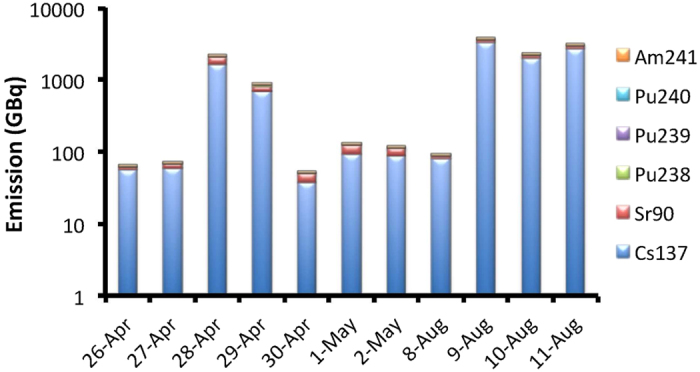
Activity levels (GBq) of daily emissions of ^137^Cs, ^90^Sr, ^238^Pu, ^239^Pu, ^240^Pu and ^241^Am from the fires of 2015 [MS-Excel. Microsoft Excel for Mac 2011 version 14.5.9. (2015) Available at: https://www.microsoft.com/en-us/download/details.aspx?id=50361 (Accessed: 17th December 2015)].

In order to assess these releases from a radiological point of view, these numbers are directly translated into radioactivity releases using the specific activity^15^. The specific activity of a radionuclide is inversely proportional to its atomic weight and its half-life. Hence, ^137^Cs and ^90^Sr are more radioactive than ^238^Pu, ^239^Pu, ^240^Pu and ^241^Am; this is shown by the respective release rates (Fig. 1). Our calculations show that about 10.9 TBq of ^137^Cs were released, 1.5 TBq of ^90^Sr, 7.8 GBq of ^238^Pu, 6.3 GBq of ^239^Pu, 9.4 GBq of ^240^Pu and 29.7 GBq of ^241^Am were released in total for both fires in spring and summer 2015.

The evolution and fate of the radioactive plume is shown in Supplementary Video 1 for the fires in April 2015. Initially, the plume followed an easterly direction towards Moscow. After a few days, it turned to the north approaching Western Russia close to the border with Finland. Shortly after, another release from the continuous burning of the CEZ was transported to the north and reached southern Finland and Sweden and subsequently Latvia and Lithuania. In parallel, radionuclides were also transported to the east, and smaller amounts to the south, i.e., towards the Black Sea but without reaching Turkey. The aforementioned circulation only describes the more labile radionuclides ^137^Cs and ^90^Sr. The refractory transuranium elements were deposited closer to the source due to the larger particle size (3.8–4.8 μm), which leads to more effective deposition. Although surface airborne concentrations of 1–10 μBq m^−3^ occurred initially for the refractory radionuclides close to the source, they decreased rapidly with time until May 6^th^.

The respective transport of ^137^Cs, ^90^Sr, ^238^Pu, ^239^Pu, ^240^Pu and ^241^Am released from the fires in August 2015 can be seen in Supplementary Video 2. The plume was immediately transported to the east during the first four days. Thereafter, the fallout burden was divided into three parts. The first travelled to the west, across the borders with Belarus and then north, developing a cyclonic direction towards Russia, which lasted for two days. Another part of the plume was transported across Poland, Northern Germany and Denmark and ended in a big anticyclone that covered whole Scandinavia. Finally, a third part of smaller intensity moved southwards affecting the Balkan region, the Aegean Sea and Turkey. The simulated concentrations were of a similar magnitude as for the spring fires for ^137^Cs and ^90^Sr, but ^238^Pu, ^239^Pu, ^240^Pu and ^241^Am concentrations were substantially lower.

### Deposition and transport efficiency of radionuclides

**Figure 2:**
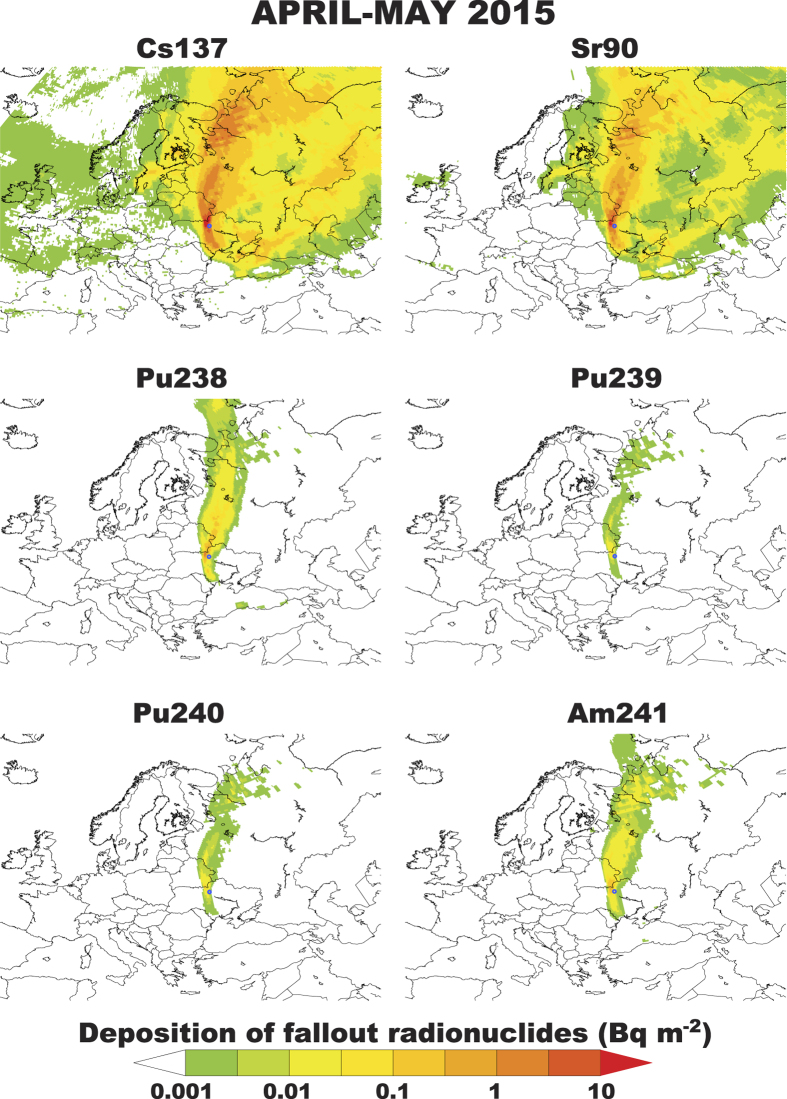
Deposition of ^137^Cs, ^90^Sr, ^238^Pu, ^239^Pu, ^240^Pu and ^241^Am over Europe from the fires occurred in spring 2015 over the CEZ. Note that the labile ^137^Cs and ^90^Sr escape from the vicinity of the CEZ [FERRET. Ferret Analysis Script Tool (FAST), Data visualisation and analysis version 6.96. (2015) Available at: http://ferret.pmel.noaa.gov/Ferret/home (Accessed: 17th December 2015)].

**Figure 3:**
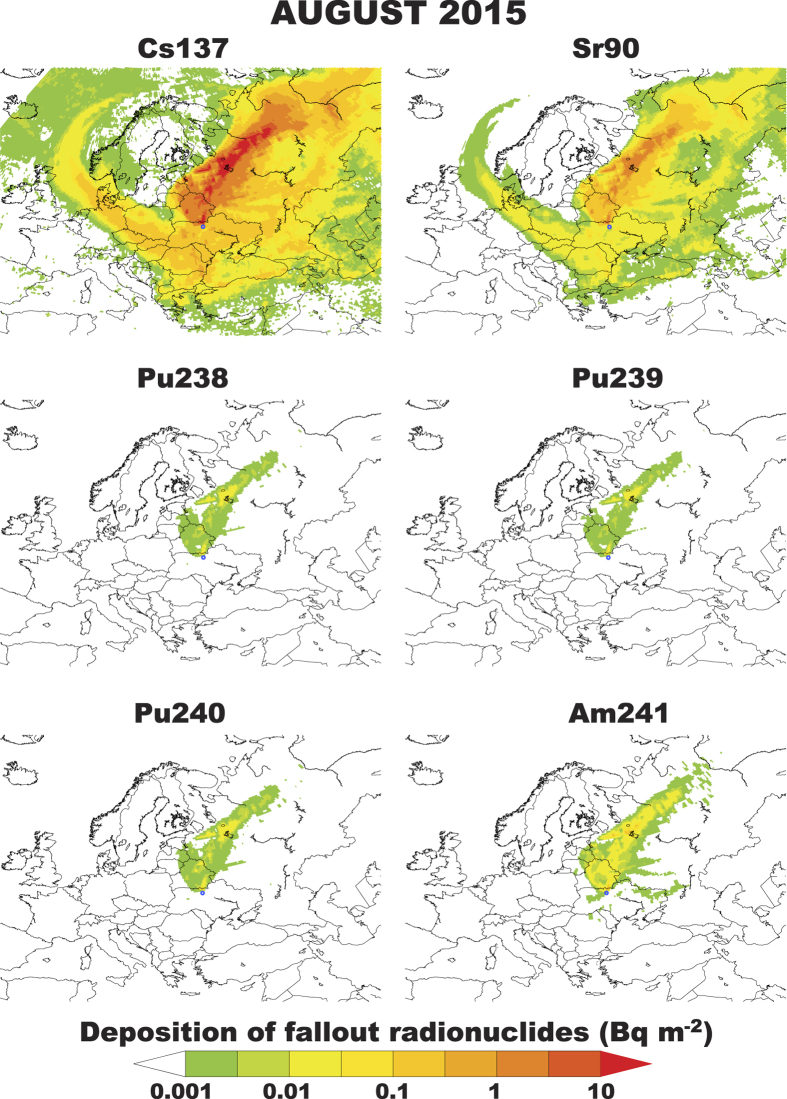
Deposition of ^137^Cs, ^90^Sr, ^238^Pu, ^239^Pu, ^240^Pu and ^241^Am over Europe from the fires occurred in August 2015 over the CEZ. Note that the labile ^137^Cs and ^90^Sr escape from the vicinity of the CEZ [FERRET. Ferret Analysis Script Tool (FAST), Data visualisation and analysis version 6.96. (2015) Available at: http://ferret.pmel.noaa.gov/Ferret/home (Accessed: 17th December 2015)].

Radionuclide deposition maps for the spring fires are shown in Fig. 2. According to our simulations, 2.1 TBq of ^137^Cs and 553 GBq of ^90^Sr were deposited over areas outside the CEZ. This amount is significantly smaller for ^238^Pu, ^239^Pu, ^240^Pu and ^241^Am (2.5, 2.0. 3.0 and 8.7 GBq) with the largest deposition occurring in the nearby areas of Southern Ukraine, Eastern Belarus and Western Russia. The rest of Europe is hardly affected from the refractory radionuclides and only ^137^Cs and ^90^Sr are deposited there, although in trace amounts. The situation was different in August 2015 with generally higher amounts exiting the CEZ (Fig. 3). The respective amounts deposited outside the CEZ were estimated to be 6.1 TBq for ^137^Cs and 613 GBq for ^90^Sr, while the respective deposition of the refractory radionuclides was 2.4 GBq of ^238^Pu, 1.9 GBq of ^239^Pu, 2.9 GBq of ^240^Pu and 9.1 GBq of ^241^Am.

To summarize the radionuclide deposition patterns, we compute the fraction of the emitted radionuclides that were deposited in certain regions around the world. This fraction can be considered as the transport efficiency of the fire-derived ^137^Cs, ^90^Sr, ^238^Pu, ^239^Pu, ^240^Pu and ^241^Am isotopes relative to the total mass emitted. These estimates are conducted by masking several geopolitical regions in Europe and calculating the amount deposited with respect to the total released. We define several regions as follows: (i) CEZ, (ii) Ukraine (excluding the CEZ), (iii) Belarus, (iv) Russia (Europe), (v) Scandinavian countries or Northern Europe (NEU: Denmark, Norway, Sweden, Iceland, Finland), (vi) Central Europe (CEU), which includes Germany, Switzerland, Poland, Czech Republic, Slovak Republic and Hungary, (vii) Western Europe (WEU: France, United Kingdom, Belgium and Holland), (viii) Eastern Europe (EEU: Estonia, Latvia, Lithuania, Belarus, Ukraine, Russia, Romania and Moldova), (ix) Southern Europe (SEU), which includes Portugal, Spain, Italy and Greece and the rest of the Balkan countries, (x) Balkan countries (Croatia, Serbia, Montenegro, Greece, Bulgaria, Albania, Slovenia) and (xi) Turkey.

The respective results are shown in Table 2, which summarizes the fate of the radioactive fallout emitted from the fires in the CEZ. During spring 2015 fires about 79% of ^137^Cs and ^90^Sr and 31% of ^238^Pu, ^239^Pu, ^240^Pu and ^241^Am were transferred and deposited outside the CEZ, mostly in Belarus (22% of ^137^Cs and ^90^Sr and 30% of ^238^Pu, ^239^Pu, ^240^Pu and ^241^Am) and Russia (50% of ^137^Cs and ^90^Sr and 35% of ^238^Pu, ^239^Pu, ^240^Pu and ^241^Am). Overall, about 93% of the labile radionuclides and 97% of the more refractory ended in Eastern Europe (EEU). Much smaller fractions of the emitted radionuclides were deposited over Scandinavia (≤0.5%), Central Europe (≤0.06%) and Southern Europe (<0.3%). Refractory radionuclides remained closer to the CEZ showing 10% higher transport efficiencies over the CEZ than labile ones.

During the fires in August 2015 about 25% of ^137^Cs and ^90^Sr deposited in the CEZ and 41% of the refractory ^238^Pu, ^239^Pu, ^240^Pu and ^241^Am. Similar to the spring fires, the rest was mainly deposited over Belarus (11% of ^137^Cs and ^90^Sr and 22% of ^238^Pu, ^239^Pu, ^240^Pu and ^241^Am) and over Russia (37% of ^137^Cs and ^90^Sr and 25% of ^238^Pu, ^239^Pu, ^240^Pu and ^241^Am). Again, the larger particles of the refractory elements were lost closer to the source, due to dry deposition, as is evident from the ratio of dry to total deposition, which is 41% for ^238^Pu, ^239^Pu, ^240^Pu and ^241^Am but only 14% for ^137^Cs and ^90^Sr. The main difference of the summer fires is that a larger fraction of the radionuclides was deposited over Central Europe (11% for ^137^Cs and ^90^Sr) and Southern Europe (5% of ^137^Cs and ^90^Sr), which mostly deposited in the Balkan countries (4.7%). Nevertheless, these amounts are radiologically insignificant, as they are distributed over large areas.

**Figure 4:**
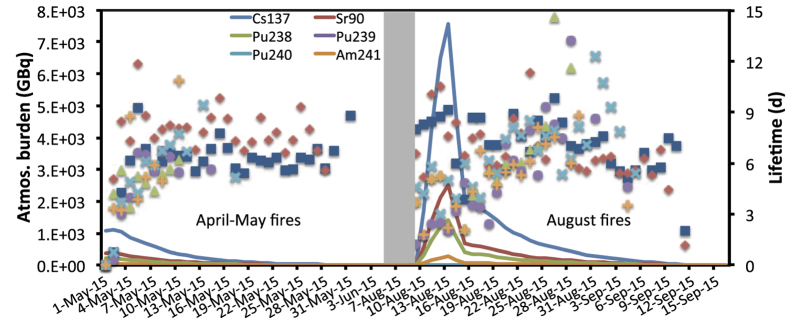
Atmospheric burden of ^137^Cs, ^90^Sr, ^238^Pu, ^239^Pu, ^240^Pu and ^241^Am emitted from forest fires in April–May and August 2015 presented as spaghetti graphs. Aerosol lifetimes (blue rectangles for ^137^Cs, red rhombus for ^90^Sr, green triangles, purple circles and light-blue ex-marks for ^238,239,240^Pu and orange cross-marks for ^241^Am) of respective radionuclides during the same two periods are presented in daily points in a secondary axis (on the right) [MS-Excel. Microsoft Excel for Mac 2011 version 14.5.9. (2015) Available at: https://www.microsoft.com/en-us/download/details.aspx?id=50361 (Accessed: 17th December 2015)].

Figure 4 depicts the atmospheric burden (amount of airborne radionuclides) of the six radionuclides emitted from the fires in spring and summer 2015 and the derived aerosol lifetimes (time that radionuclides stay in the air until their loss through deposition). The global mean aerosol-bound lifetime of ^137^Cs was estimated to be 6.8 ± 1.2 d, while for ^90^Sr it was 7.3 ± 1.5 d. The respective lifetimes of the refractory radionuclides ^238^Pu, ^239^Pu, ^240^Pu and ^241^Am were about 33% lower (4.9 ± 1.5 d, 4.5 ± 1.8 d, 5.2 ± 1.0 d and 4.5 ± 1.4 d, respectively). This partly explains why refractory elements cannot escape from the close vicinity of the CEZ in large quantities, together with the fact that refractory elements assumed to have half the emission factors of the labile ones; thus, the quantities emitted were lower by some orders of magnitude. Mean aerosol lifetimes from global models are typically in the range of 3–7 days^16^,^17^,^18^, very similar to our estimations (4.5–7.3 d). The exponential decrease of the atmospheric burden of the six radionuclides (Fig. 4) was used to calculate their effective half-lives. These were found to be 3.2–4.7 d for ^137^Cs and ^90^Sr and slightly smaller for the refractory radionuclides ^238^Pu, ^239^Pu, ^240^Pu and ^241^Am (2.5–3.3 d), which is about half of our global lifetime calculations and similar to other findings^19^,^20^.

### Dosimetric assessment

**Figure 5:**
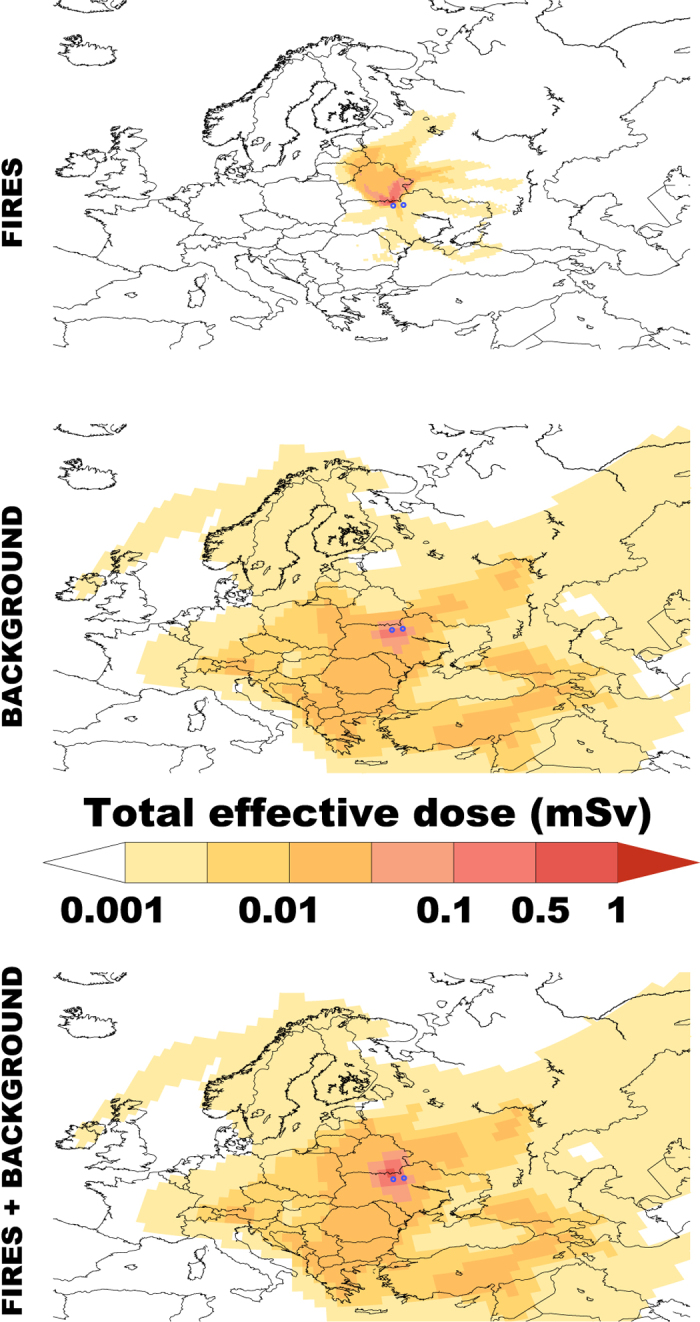
Total effective dose over Europe after the fires in spring and summer 2015 in the CEZ. The upper panel shows the additional effective dose from inhalation, air-submersion and deposition of ^137^Cs resuspended by the fires (0.25° × 0.25°). The middle panel shows the effective dose over Europe due to background radiaition from the deposition of ^137^Cs after the Chernobyl accident in 1986 and assuming an effective half-life of 10 years^22^ [FERRET. Ferret Analysis Script Tool (FAST), Data visualisation and analysis version 6.96. (2015) Available at: http://ferret.pmel.noaa.gov/Ferret/home (Accessed: 17th December 2015)]. The lower panel depicts the total effective dose (1° × 1°) including the Chernobyl background.

The total effective doses over Europe from fires in the CEZ for the year 2015 are shown in the upper panel of Fig. 5. In the middle panel, the total effective dose from the background radiation from the Chernobyl–remaining deposition of ^137^Cs is given. The latter was estimated using the deposition data presented in www.radio.nilu.no. The data have been cross–validated with the Chernobyl simulations of Evangeliou *et al.*^21^ and applying an effective half-life of 10 years from Bergan^22^.

Effective doses are high near the source (CEZ) and decline significantly with increasing distance. Although the majority of ^137^Cs (80%) was deposited outside CEZ, most of it remained in the nearby areas of Belarus and Russia as the CEZ lies between the borders of Ukraine, Belarus and Russia. Internal exposure pathway of inhalation holds the largest share of the effective dose (63%), while deposition and air-submersion contribute 25 and 12%, respectively. This means that during future years this dose will be much lower due to the loss of airborne ^137^Cs, although ^137^Cs in the air of the CEZ from ground resuspension cannot be assessed. The 2015 fires in the CEZ almost doubled the total effective dose close in the contaminated forests as seen in Fig. 5 (lower panel), resulting in a dose above 1 mSv y^−1^ (in the CEZ only). Values were far below this threshold in the rest of Europe (<0.005 mSv in most of Europe) basically caused by the Chernobyl background. The 1 mSv y^−1^ threshold is the annual effective dose limit established for members of the public in planned exposure situations (limits do not apply to existing or emergency exposure situations) as stated in WHO^23^. To put the estimated effective dose levels into perspective, it is noted that external exposure from cosmic rays during transoceanic flights gives an annual average effective dose of 0.39 mSv, terrestrial radiation (indoors and outdoors) gives 0.48 mSv on average, radon inhalation exposure varies globally between 0.2 and 10 mSv, while effective doses from diagnostic radiological medical procedures range from 0.005 (dental X-ray) to 15 mSv (abdomen and pelvis CT scan)^23^. Hence, the estimated dose levels over Europe (apart for the close vicinity of the CEZ) are far below to the typical effective dose resulting from external exposure to a medical X-ray (chest X-ray: 0.02 mSv, Skull X-ray: 0.06 mSv)^23^, when including the background of the Chernobyl accident. When only fire-resuspended radioactivity is accounted for, the effective dose is far below 0.001 mSv for most of Europe, with the exception of Ukraine, Belarus, Western Russia and some of the Baltic countries (Fig. 5).

**Figure 6:**
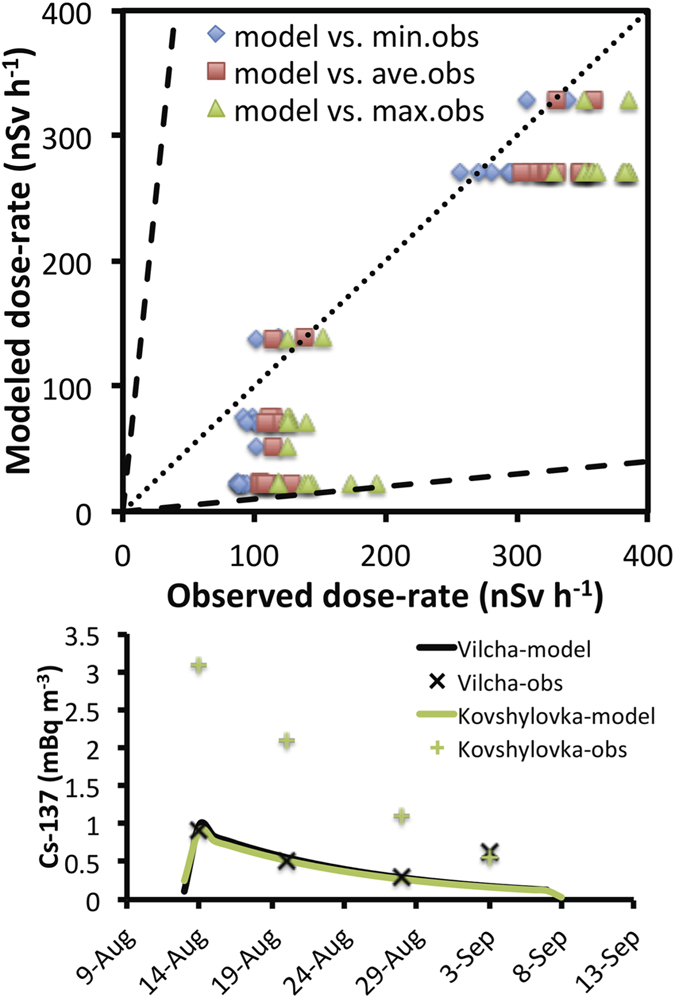
Comparison of modeled and observed equivalent dose rates and radionuclide concentrations. Upper panel: Scatter-plot of modeled equivalent dose-rates versus observations obtained from six stations of the automated radiation monitoring system (ARMS) during the summer 2015 fires in the CEZ. Modeled dose-rates are compared with minimum (blue), mean (red) and maximum (green) observed values. Bottom panel: Time-series of modeled surface activity concentrations of ^137^Cs in Vilcha (black) and Kovshylovka (green) villages compared with observations [MS-Excel. Microsoft Excel for Mac 2011 version 14.5.9. (2015) Available at: https://www.microsoft.com/en-us/download/details.aspx?id=50361 (Accessed: 17th December 2015)].

It was previously mentioned that background radiation is monitored by ARMS in continuous mode at 39 stations around the CEZ. Near the fire area of summer 2015, six ARMS stations are located (Vilcha, Dibrova, Stara Rudnia, Polisske, Maksymovichi and Marajanivkla villages). Equivalent gamma dose rate observations became available^1^ and were used for a comparison with instantaneous external equivalent dose rates calculated from the FLEXPART model output for individual days during the summer 2015 fires^24^. The comparison is shown in the top panel of Fig. 6. Different colours indicate comparison with minimum (blue), average (red) and maximum (green) detected dose-rates, as ARMS provide dose rates measurements every 2 minutes. The model managed to capture well the observed dose-rates in most stations, considering the assumption made on the simulations (e.g. emission factors). Some model overestimations occurred during the very first days at stations close to the fire, but they were below a factor of two.

Air activity concentrations of ^137^Cs in the vicinity of a fire were determined from 14 August to 10 September 2015 (weekly) close to Vilcha and Kovshylovka villages. Levels of surface activity concentration of ^137^Cs significantly decreased with time after the fires were extinguished. Near Kovshylivka village, ^137^Cs concentrations in the air were above the established reference safety levels (210 μBq m^−3^), namely “basic reference levels, exemption levels and action levels of radioactive contamination in the exclusion zone and zone of unconditional (mandatory) resettlement”. Accordingly, the personnel involved in fire-fighting activities in the fire area used respiratory protective equipment and underwent monitoring of ^137^Cs concentration in the body. Comparison of our model results to measured levels of surface ^137^Cs is shown in Fig. 6 (bottom panel). The model resolution (0.25° × 0.25°) allowed distinguishing surface concentrations in the two different villages. In Vilcha, the model reproduced measurements of surface ^137^Cs extremely well, also capturing the decreasing trends of radiation. This was not the case in Kovshylovkam, where the model underestimated observations by a factor of 2.5.

The differences in modeled surface activity concentrations and estimated dose-rates with observations are multifold. For instance, the poor model resolution plays a major role in distinguishing concentration levels in the two villages. Another reason is the assumptions on the emission factors used in the model. The underestimated concentrations in Kovshylovkam may imply that a larger emission factor for ^137^Cs should be used. On the contrary, the concentrations were much higher in other adjacent pixels, where data were not available for comparison. Finally, a constant emission profile for the releases was used in the simulations (PRMv2), whereas it is well–established that the altitude of the emission varies according to the fire intensity. Nevertheless, the number of available measurements is very small and no definitive conclusions about the performance of the model can be drawn. However, it is noteworthy that both in Vilcha and Kovshylovkam the measured concentrations of ^137^Cs exceeded the reference level (0.21 mBq m^−3^) and reached up to 1 order of magnitude higher.

## Discussion

A basic concern when assessing radioactive releases is whether or not they are dangerous enough to require safety measures for the public. For this reason, the International Nuclear and Radiological Event Scale (INES)^25^ has been established. INES includes seven levels: Levels 1–3 are called “incidents” and Levels 4–7 “accidents”. The scale is designed so that the severity of an event is about ten times greater for each increase in level on the scale. Nevertheless, INES has not been designed for forest fire releases and, hence, its use may not be suitable in the present case. It is only used as a qualitative estimate of the release severity with respect to nuclear accidents and it should not be given significant weight. The highest four levels on the scale (Levels 4–7) include in their definition the quantity of activity released, defining its size by its radiological equivalence to a given number of TBq of ^131^I. Depending on the nature of the atmospheric releases (direct by a NPP or from other generic radiation sources) baseline factors characteristic for each of the released radionuclides are used (D2 factors). These factors are 20 TBq for ^137^Cs, 1 TBq for ^90^Sr and 0.06 TBq for ^238–240^Pu and ^241^Am.

The releases of ^137^Cs, ^90^Sr, ^238^Pu, ^239^Pu, ^240^Pu and ^241^Am after the 2015 fires were 10.9 TBq, 1.5 TBq, 7.8 GBq, 6.3 GBq, 9.4 GBq and 29.7 GBq, respectively. Summing up the D2 factors of the released radionuclides gives a total of 21.24 TBq, whereas the total released amount of radioactivity is 12.45 TBq. The release is about half of the D2 factor, which corresponds to “Level 3” on INES. This is translated into a “serious incident”, in which non-lethal deterministic effects are expected from radiation. For comparison, it should be noted that only Chernobyl and Fukushima were rated as “Level 7”, the Kyshtym disaster at Mayak Chemical Combine (MCC) in the ex-Soviet Union was assessed as “Level 6”, the Windscale fire (United Kingdom) and Three Mile Island accident (USA) as “Level 5”, and Sellafield (United Kingdom) as “Level 4”, while a number of other releases of insignificant extent was also reported^26^,^27^. Again, it is necessary to point out that the INES was not designed for such kind of releases and its use might be abusive.

The current political instability in Ukraine and the lack of financial resources certainly affects the fire-fighting capacity in the CEZ. Significant fire-fighting infrastructure is lacking in the CEZ and renewal of the fire crew and modernization of the fire-fighting equipment remain only political promises at the moment. Fire prevention has not been given enough attention in the region. The absence of the basic components of fire prevention in CEZ, such as fire detection and early warning systems, has already led to a series of catastrophic wildfires (e.g. in 1992). Fire hazards have been increasing steadily in the CEZ and ignition sources are present throughout the territory of the CEZ during the entire fire season mainly because local villagers, CEZ staff, and illegal visitors conduct agricultural burns. According to official statistics, about 1100 small and medium size wildfires occurred during 1993–2010 in the CEZ, including the most contaminated zones^28^.

**Figure 7:**
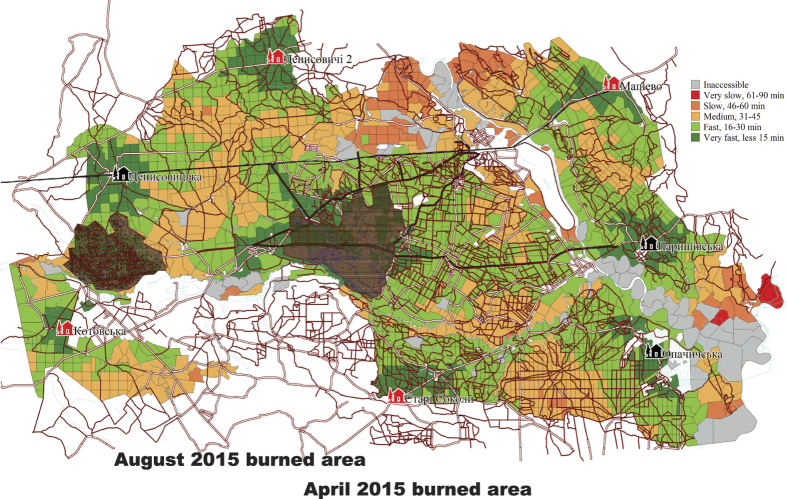
Zones with different response times needed for fire crews to reach certain parts of CEZ in case of fire (gray – unreachable, orange and red – unsatisfactory – from 45 to 90 minutes). Note: lack of forest roads can delay responses. The four black markers north, south, west and east denote the available fire stations, whereas values up on the right denote minutes to access each forest area within CEZ. The burned areas of April and August 2015 are masked (dark shadow) [GIMP. GNU Image Manipulation Program version 2.8.16. (2015) Available at: https://www.gimp.org (Accessed: 17th December 2015)].

Moreover, fire-fighting staff has been cut by almost 40% over the past two decades and the existing fire detection systems cover only 30–40% of the CEZ. Most of the heavy fire-fighting equipment is at least 10 years old, out-dated, and not reliable in dealing with fire emergencies. The number of fire stations, fire crew, and fire engines is much smaller than what would be expected for the area they cover, while only 20–30% of the required prevention measures in the CEZ are executed annually due to lack of funding and the dramatic reduction of the forest road network available for fire engines. Thus, there are many parts of the CEZ that are practically inaccessible by fire-fighters or require more than 45 minutes to reach them (Fig. 7).

Such problems with infrastructure and personnel greatly increase the probability of a future fire that gets out of control. The risk will be enhanced by the predicted future climatic warming of the region that will lead to increased frequency and severity of drought^6^. There were three large fires in the CEZ in 2015 (a smaller fire in June was not included in the present study due to lack of information) demonstrating the importance of the area in terms of a secondary radioactive contamination hazard for Europe. Steps forward to improve safety should start with the employment of fire crew personnel who will execute the prevention measures and take part in the fire-fighting process.

## Methods

### Defining active fires and burned area in the CEZ

Remote sensing has been useful for delineating fire perimeters, characterizing burn severity and planning post-fire restoration activities in different regions. The use of satellite imaging is particularly important for fire monitoring in the CEZ. Due to the prevailing high radioactive contamination, it was impossible to implement reliable terrestrial fire observations on large areas that still remain difficult to access. Fire perimeters and burn severity were extracted from Landsat images by applying the differenced Normalized Burn Ratio (dNBR)^29^:


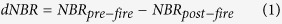


Normalized burn ratio for pre- (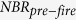) and postfire (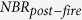) Landsat 8 OLI (Operational Land Imager) images can be calculated using near- and shortwave infrared bands (bands 5 NIR and 7 SWIR 2 at 0.85–0.88 μm and 2.11–2.29 μm, respectively):


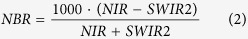


There are a lot of examples in the literature of application of dNBR index to assess the impact of fires in the pine forests of Northern and Western USA^29^,^30^ as well as in other environments^31^,^32^.

The burned severity mosaics were created using Landsat 8 OLI images. For both fires, pre– and post–fire images were used to create cloudless mosaics (Supplementary Table S1). This procedure was performed after conversion of the haze values of the satellite images to Top of Atmosphere (TOA) reflectance^33^. The aforementioned step is crucial for simultaneous processing of multi-seasonal satellite images that cover large areas.

We have also created pre-fire mosaics to map burn severity for the April and August 2015 fires. This was implemented using the Maximum Value Composite procedure (MVC)^34^. The method only selects free pixels from each band that also have higher value of Normalized Difference Vegetation Index (NDVI). The same algorithm was used for post–fire images as well, but pixels having minimum NBR were only selected. With this method, we removed the influence of smoke on the image processing, and took into account the final ecosystem damage caused by fire. Additional classification rules were imposed to map a more precise burn severity, due to the sensitivity of NBR to changes in vegetation and soil moisture. A 500 m buffer zone around the manually delineated fire perimeters was applied and all areas outside were classified as unburned. We have used common dNBR severity levels^29^, which are presented in Supplementary Table S2 and Supplementary Fig. S1.

In order to reconstruct fire dynamics in the CEZ during April 2015, NASA’s (National Aeronautics and Space Administration) Moderate Resolution Imaging Spectroradiometer (MODIS) real time data have been downloaded from LANCE (Land, Atmosphere Near real-time Capability for EOS)^35^. Coordinates of fire locations (hot spots) were downloaded from FIRMS (Fire Information for Resource Management System)^36^. These data accompanied by MODIS multispectral images allowed delineating fire perimeters for every day of the spring fire events (Supplementary Fig. S2). The same analysis was conducted for the August 2015 fires in the CEZ, but hot spots could not be detected due to dense cloud cover in the area.

### Levels of deposition, emission factors and emission height

Pre-fire deposition densities of ^137^Cs, ^90^Sr, ^238^Pu, ^239^Pu, ^240^Pu and ^241^Am in the burned area are needed in order to estimate the radionuclide resuspension by the fires. We used measurements from Kashparov *et al.*^10^,^11^, which have been validated using observation maps^13^. Furthermore, they are freely available for research purposes and can be obtained upon request. They are shown in Supplementary Fig. S3 as Box & Whisker plots.

No measurements of radioactivity in biomass from the respective burned areas in the CEZ were available except for a few scattered samples. It has been reported that a minimum of at least 20% of labile radionuclides will be redistributed in the atmosphere after a fire, no matter whether they are deposited in the soil^3^ or biomass/vegetation^37^,^38^,^39^. More specifically, the emission factors of labile radionuclides range from 20% in soil^3^ up to 70–100% in vegetation for intensive wildfires^37^,^40^. As for the refractory radionuclides, to our knowledge, no measurements of emission factors for biomass burning exist. However, we expect that the emission factor will be lower and at least half (10%) of the value used for the labile radionuclides. In summary, we assumed that in each burned pixel 20% of the deposited amount of the labile radionuclides (^137^Cs, ^90^Sr) and 10% of the refractory ones (^238^Pu, ^239^Pu, ^240^Pu and ^241^Am) were released to the atmosphere. To quantify the emission amounts for the purpose of dispersion modelling, the spatial deposition density of radionuclides (Bq m^−2^) in the area (Supplementary Fig. S3 and http://radio.nilu.no) was multiplied with the burned area (m^2^) estimated from Landsat 8 OLI images and applying an emission factor of 20% for ^137^Cs and ^90^Sr and 10% for ^238^Pu, ^239^Pu, ^240^Pu and ^241^Am.

Injection heights into the atmosphere of the emitted smoke were simulated with the Plume Rise Model (PRM) presented in Paugam *et al.*^41^. The model (hereafter referred to as PRMv2) is a further development of the PRM of Freitas *et al.*^42^,^43^ and has already been used in previous studies of Chernobyl’s fire events^6^,^8^. The model gives a profile of smoke detrainment for every single fire, from which two metrics are extracted: (i) a detrainment layer (i.e. where the detrainment rate is >50% of its global maximum) and (ii) an injection height (InjH, the top of the detrainment layer). Supplementary Fig. S4 shows for all fires detected by the MODIS fire product^44^ during the two time periods (26 April–2 May 2015 and 9–14 August 2015): (a) the horizontal distribution of the integrated median InjH median (Supplementary Fig. S4, left panels), (b) single fire events with a modeled plume height above 5 km (scattered circles in left panels of Supplementary Fig. S4), and (c) the vertical distribution of the emissions × conversion factor, see Kaiser *et al.*^45^) integrated over all longitudes (see Supplementary Fig. S4, right panels). For the two time periods, fires that occurred in the highly contaminated zones close to the CNPP show maximum injection heights around 5 km, but according to PRMv2 the majority of the emissions (85%) remained within the Planetary Boundary Layer (PBL). This is quite typical for the region, according to previous observations of injection heights for boreal forest fire emissions from the satellite observations^46^,^47^. For modelling the dispersion of the radioactive smoke, we assumed that 85% of the emissions were homogenously mixed in the PBL and the remaining 15% were uniformly distributed between the top of the PBL and 5km.

### Atmospheric modelling

Emissions were fed to the Lagrangian particle transport model FLEXPART (Flexible Particle Dispersion Model)^48^,^49^, which simulated the transport and deposition of the fire-redistributed radionuclides. This was implemented by running in forward mode from April 1^st^ to May 31^st^, and from August 1^st^ to November 30^th^, 2015. Simulations used the emissions of ^137^Cs, ^90^Sr, ^238^Pu, ^239^Pu, ^240^Pu and ^241^Am as defined in the previous section. They were driven by 3-hourly 1° × 1° operational analyses from the European Centre for Medium Range Weather Forecast^50^. The resolution of the modeled concentration and deposition fields was set to 0.25° × 0.25° in a global domain. This resolution should be sufficient to capture the spatiotemporal variability of radionuclide concentrations in the vicinity of the CEZ, in the adjacent countries and on somewhat larger scales (i.e., beyond some 100 km from the source area / CEZ).

Cesium-137 and ^90^Sr are typically attached to sub-micron particles (<1 μm, e.g. Garger *et al.*^51^, Masson *et al.*^52^, Ooe *et al.*^53^), whereas the refractory ^238^Pu, ^239^Pu, ^240^Pu and ^241^Am radionuclides are typically found in coarse mode (1–10 μm) particles. Plutonium isotopes and ^241^Am have been reported to reside in the coarse mode characterized by Activity Median Aerodynamic Diameters (AMAD) of 3.8–4.8 μm for the vast majority of cases^51^. Therefore, the diameter in FLEXPART has been set to 4 μm for plutonium and ^241^Am particles and to 0.6 μm for ^137^Cs and ^90^Sr with an assumed particle density of 2500 kg m^−3^.

### Aerosol lifetimes and half-lives

The aging of the studied aerosol species, and how far they may be transported after a release, is mainly a result of the different lifetimes of the labile and refractory radionuclides. In the atmosphere, the mass balance of a species can be expressed as follows:


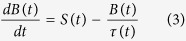


where 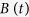 is the atmospheric burden, 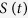 is the release rate and 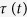 is the removal time over a given time-step. If one assumes equilibrium between release rate and deposition, the mean lifetime (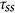) will be:


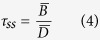


where 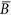 and 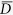 are the mean atmospheric burden and deposition over a given period^54^.

When dealing with radioactive species, the so-called effective half-life is frequently used. The latter combines two loss parameters of a radionuclide present in the atmospheric aerosol; the ecological and the radioactive or physical decay. The ecological half-life is defined as the period of time it takes for radioactive burden to decrease by half, due to processes other than radioactive decay (e.g. wet and/or scavenging). The mathematical expression of the atmospheric processes is given below^18^:


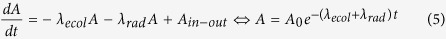


where *A* is the mass of a given radionuclide at time 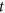 (d), 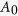 is the initial amount at zero time, 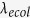 is the ecological decay constant of the radionuclide (d^−1^), 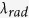 is the radioactive (or physical) decay constant (e.g. for ^137^Cs it is 6.29 × 10^−5^ d^−1^), 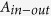 is the portion that enters or exits a hypothetical box (it is neglected since we estimate global half-lives) and 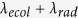 is the effective decay constant 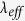. Hence, the exponential decrease of the atmospheric burden of a given radionuclide after the end of the emissions can be used to quantify the effective half-life defined as:


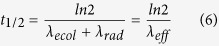


### Dosimetric calculations

Considering that dosimetric quantities are needed to assess human radiation exposure in a quantitative way, the internal and external dose-rates from all possible pathways are calculated for the year 2015. Dose-rates are calculated for gamma-emitters (^137^Cs only) from deposition and air-submersion (external pathways) and inhalation (internal pathway). The pathway of ingestion of food and water is omitted due to the lack of available data.

The dosimetric scheme for the calculations of human dose rates was adopted from the WHO^23^ report on Fukushima for adults and includes the most updated approaches on dose calculations. The effective dose-rate from deposition is given by the following equation:


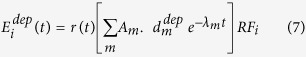


where the summation index m is for each of the deposited radionuclides, 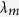 is the decay constant of radionuclide m (0.023 y^−1^ for ^137^Cs), 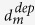 is the dose rate coefficient from surface activity density to kerma rate in free air (^137^Cs + ^137m^Ba: 1.2 × 10^−8^ Sv per Bq m^−2^), 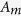 is the surface activity density of radionuclide m on the ground (Bq m^−2^), 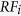 is a reduction factor based on occupancy of the population (assumed to be 0.6 for adults) and 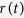 is a time dependent attenuation factor that accounts for radionuclide penetration in the soil (for the first 6 months of our simulations the minimum value is 0.925). The effective dose-rate from air-submersion is calculated as follows:


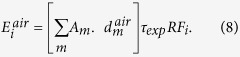


where 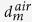 is the dose rate coefficient from a semi-infinite volume source in air to kerma in free air for a height of 1 m above ground due to uniform distribution of radionuclide m in the air (^137^Cs + ^137m^Ba: 1.3 × 10^−4^ nSv h^−1^ per Bq m^−3^) and 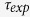 is the exposure time (we assume it is 24 h accounting for a reduction due to occupancy). The effective dose from air–submersion is integrated over the period where concentrations are above zero. Finally, the effective dose from inhalation of radioactive materials was calculated according to:


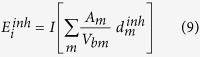


where *I* is the breathing rate for population group (for adults: 2.57 × 10^−4 ^m^3^ s^−1^), 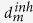 is the effective dose inhalation coefficient for adults and for radionuclide m (^137^Cs + ^137m^Ba: 4.6 × 10^−9 ^Sv per Bq) and 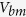 is bulk deposition velocity of radionuclide m for particular weather and surface conditions (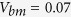 m s^−1^). Then the effective dose-rate is integrated over 2015 to give the total effective dose.

Equivalent dose rate (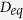 in nSv h^−1^) from air–immersion is calculated from absorbed dose rate (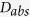 in nGy h^−1^) applying a weighting factor for gamma rays (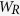 = 1):


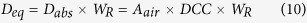


where 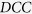 is the conversion factor in nGy h^−1^ per Bq m^−3^.

## Additional Information

**How to cite this article**: Evangeliou, N. *et al.* Resuspension and atmospheric transport of radionuclides due to wildfires near the Chernobyl Nuclear Power Plant in 2015: An impact assessment. *Sci. Rep.* **6**, 26062; doi: 10.1038/srep26062 (2016).

## Supplementary Material

Supplementary Video S1

Supplementary Video S2

Supplementary Information

## Figures and Tables

**Table 1:** Land cover types in the burned area of April–May 2015.

	Land use categories	Area (hectares)		Land use categories	Area (hectares)
April 2015	Former agricultural lands	4,464	August 2015	Natural forests	2,998
	Artificial forests	2,644		Plantations	1,555
	Natural forests	1,839		Former agriculture lands	1,106
	Swamps	1,399		Swamps	122
	Young forest plantations	200		Wildlife land	23
	Reclamation channels	120		Mixed natural / plantations forests	13
	Forest glades	68		Ameliorative channels	12
	Power lines	55		Forest roads	9
	Past years burned forests	45		Lakes	7
	Woodlands	18		Sands	6
	Sands	9		Openings	6
	Buildings and structures	8		Forest Kvartals (blocks) borders	4
	Fire breaks	8		Cut areas	3
	Forest roads	4		Electric Power lines	3
	Deceased forests	<1			
	Lakes	<1			
	**Total**	**10,882**		**Total**	**5,867**

In both cases more than half of the area corresponds to forests, as they persist with more than 70 in the CEZ.

**Table 2:** Transport efficiencies (%) of radionuclides emitted from fires in the CEZ over European areas in spring and summer 2015.

	Cs137	Sr90	Pu238	Pu239	Pu240	Am241
April 2015						
CEZ	21	20	30	30	31	31
Ukraine (excluding CEZ)	0.0005	0.0009	0.0111	0.0006	0.0001	0.0006
Belarus	22	22	30	30	31	31
Russia (Europe)	50	50	33	35	36	35
Balkan	0.02	0.02	0.0004	0.0006	0.0008	0.002
Turkey	0.014	0.06	0.01	0.005	0.01	0.05
NEU (Scandinavia)	0.5	0.5	0.4	0.2	0.2	0.2
CEU	0.05	0.06	0.001	0.002	0.003	0.008
WEU	0.1	0.1	0.003	0.001	0.003	0.01
EEU	93	93	95	95	99	98
SEU	0.02	0.03	0.0007	0.0003	0.0008	0.004
Europe (total)	**93**	**93**	**95**	**95**	**99**	**98**
August 2015						
CEZ	25	26	41	41	41	41
Ukraine (excluding CEZ)	0.008	0.008	0.004	0.004	0.004	0.004
Belarus	11	11	22	21	22	22
Russia (Europe)	37	37	25	25	26	26
Balkan	4.7	4.7	0.2	0.2	0.2	0.2
Turkey	0.6	0.6	0.04	0.04	0.04	0.04
NEU (Scandinavia)	0.03	0.03	0.006	0.006	0.006	0.006
CEU	12	11	0.8	0.8	0.8	0.8
WEU	0.2	0.2	0.04	0.04	0.04	0.04
EEU	80	80	99	99	99	99
SEU	5.1	5.1	0.2	0.2	0.2	0.2
Europe (total)	**92**	**91**	**100**	**100**	**100**	**100**
