# Supplementary material for: Resuspension and atmospheric transport of radionuclides due to wildfires near the Chernobyl Nuclear Power Plant in 2015: An impact assessment
Source: Sci Rep. 2016 May 17;6:26062. doi: 10.1038/srep26062 (PMC4869032; doi:10.1038/srep26062)
Supplement: Supplementary Information [file srep26062-s3.pdf]

Resuspension and atmospheric transport of radionuclides due to wildfires near the Chernobyl Nuclear Power Plant in 2015: An impact assessment

N. Evangeliou[1] *, S. Zibtsev[2], V. Myroniuk[2], M. Zhurba[2], T. Hamburger[1], A. Stohl[1], Y. Balkanski[3], R. Paugam[4], T. A. Mousseau[5], A. P. Møller[6], S. I. Kireev[7]

[1]Norwegian Institute for Air Research (NILU), Department of Atmospheric and Climate Research (ATMOS), Kjeller, Norway.

[2]National University of Life and Environmental Sciences of Ukraine, Kiev, Ukraine.

[3]CEA-UVSQ-CNRS UMR 8212, Institut Pierre et Simon Laplace, Laboratoire des Sciences du Climat et de l'Environnement (LSCE), L'Orme des Merisiers, F-91191 Gif-sur-Yvette Cedex, France.

[4]King's College London, London, United Kingdom.

[5]Department of Biological Sciences, University of South Carolina, Columbia, SC 29208, USA.

[6]Laboratoire d'Ecologie, Systématique et Evolution, CNRS UMR 8079, Université Paris-Sud, Bâtiment 362, F-91405 Orsay Cedex, France.

[7]Deputy General Director of the State Enterprise "Chernobyl Special Kombinat", Chernobyl city, 6 Shkolna street, Ukraine.

* Corresponding author: N. Evangeliou (Nikolaos.Evangeliou@nilu.no)

SUPPLEMENTARY VIDEO LEGENDS, FIGURES AND TABLES

Video S 1. Plume evolution of the radionuclides emitted after the forest fires in Chernobyl in April – May 2015 [56]. The video depicts surface activity concentrations (μBq m^{-3}) of the radionuclides transported over Europe [FERRET. Ferret Analysis Script Tool (FAST), Data visualisation and analysis version 6.96. (2015) Available at: http://ferret.pmel.noaa.gov/Ferret/home (Accessed: 17th December 2015)].

Video S 2. Plume evolution of the radionuclides emitted after the forest fires in Chernobyl in August 2015 [56]. The video depicts surface activity concentrations (μBq m^{-3}) of the radionuclides transported over Europe [FERRET. Ferret Analysis Script Tool (FAST), Data visualisation and analysis version 6.96. (2015) Available at: http://ferret.pmel.noaa.gov/Ferret/home (Accessed: 17th December 2015)].

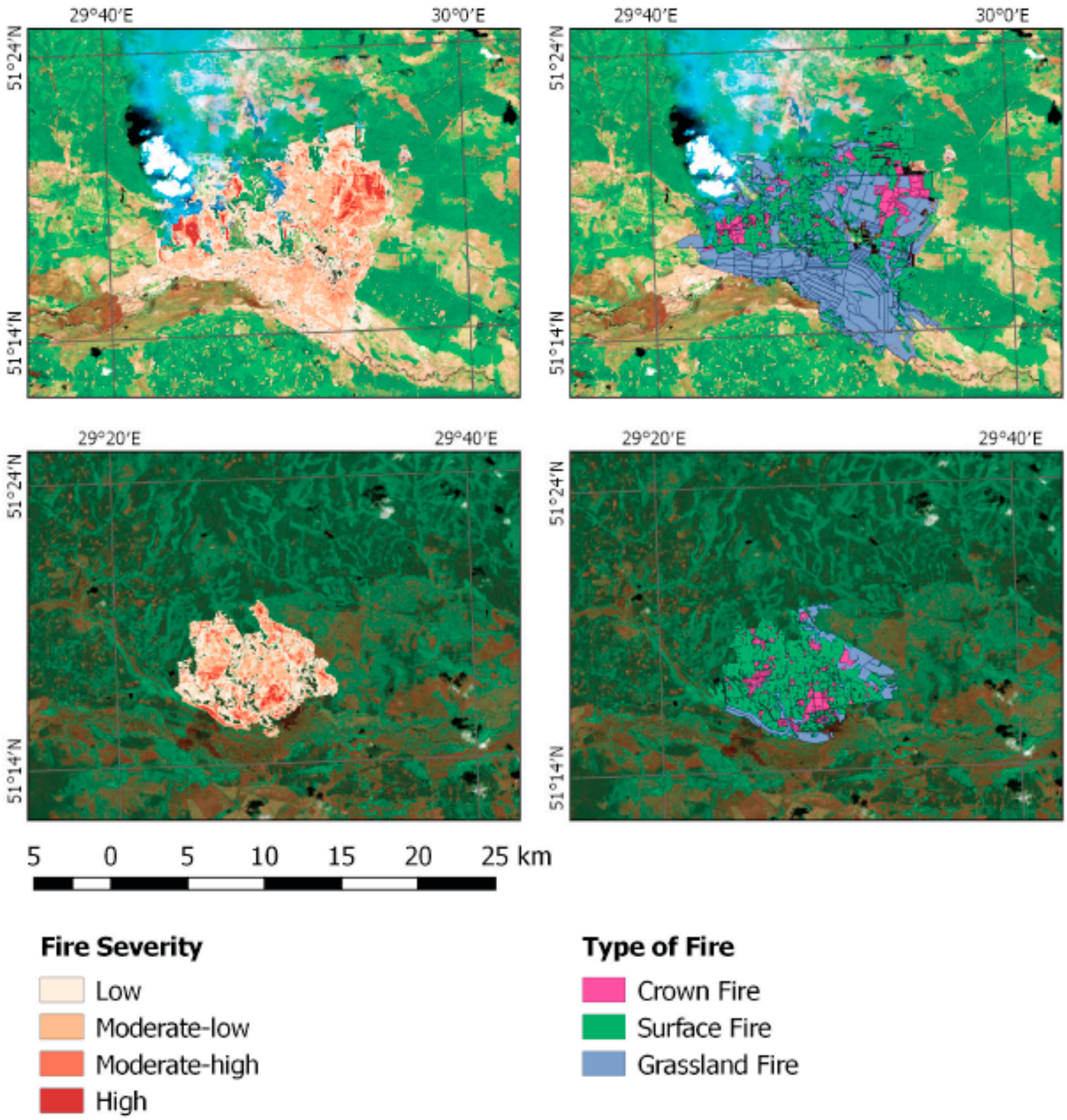

Fig. S 1. Burned area classification according to Landsat 8 OLI data for fires burning in spring (upper panels) and summer (lower panels) 2015. The left column shows fire severity and the right shows the type of these fires [R. *The R Project for Statistical Computing version 3.2.3*. (2015) Available at: https://www.r-project.org (Accessed: 17th December 2015)].

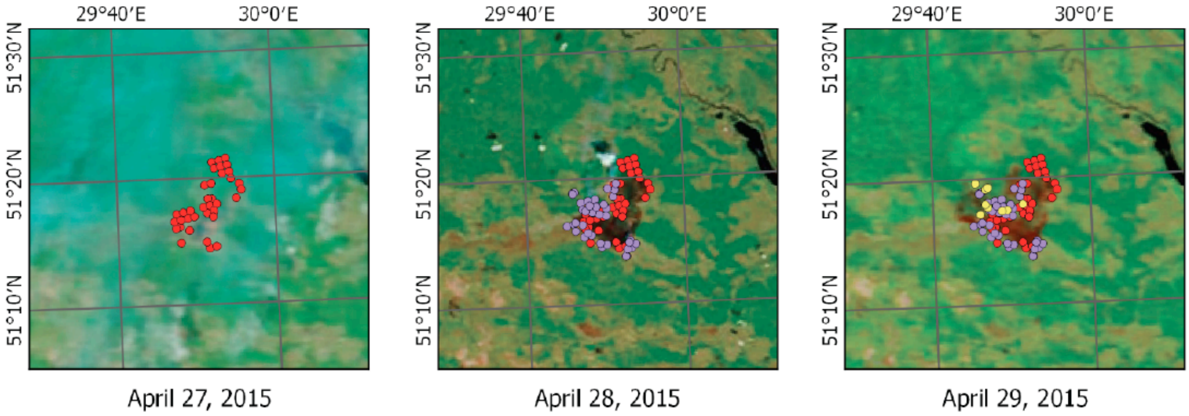

Fig. S 2. Fire dynamics in the Chernobyl exclusion zone for the April fires according to MODIS data. Dots show location of hot spots, while the active fires of April 27th are depicted in red, of April 28th in purple and of April 29th in yellow. Dark areas in the background of the hot spots denote the burned area [R. *The R Project for Statistical Computing version 3.2.3*. (2015) Available at: https://www.r-project.org (Accessed: 17th December 2015)].

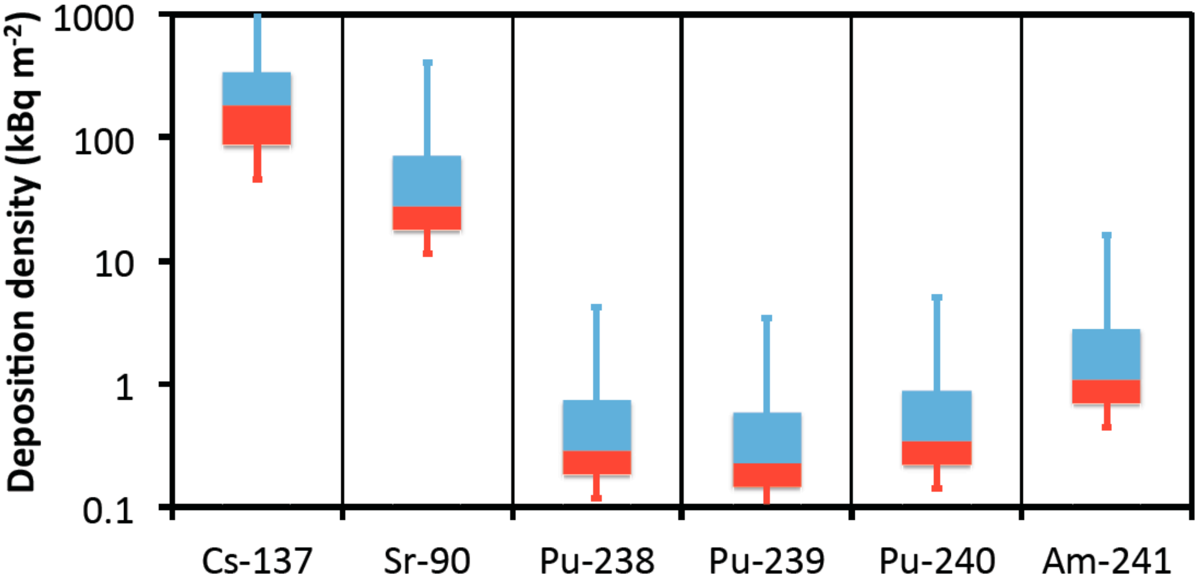

Fig. S 3. Deposition densities (number of observations = 48,781) of ^{137}Cs, ^{90}Sr, ^{238}Pu, ^{239}Pu, ^{240}Pu and ^{241}Am in longitudes 29.3°E–30.0°E and latitudes 51.2°N–51.6°N as recorded by the Ukrainian authorities prior to the spring 2015 fires. The plots show the minimum value, the 25th percentile (in red), the median, the 75th percentile (in blue) and the maximum. The data are stored in http://radio.nilu.no [MS-Excel. Microsoft Excel for Mac 2011 version 14.5.9. (2015) Available at: https://www.microsoft.com/en-us/download/details.aspx?id=50361 (Accessed: 17th December 2015)].

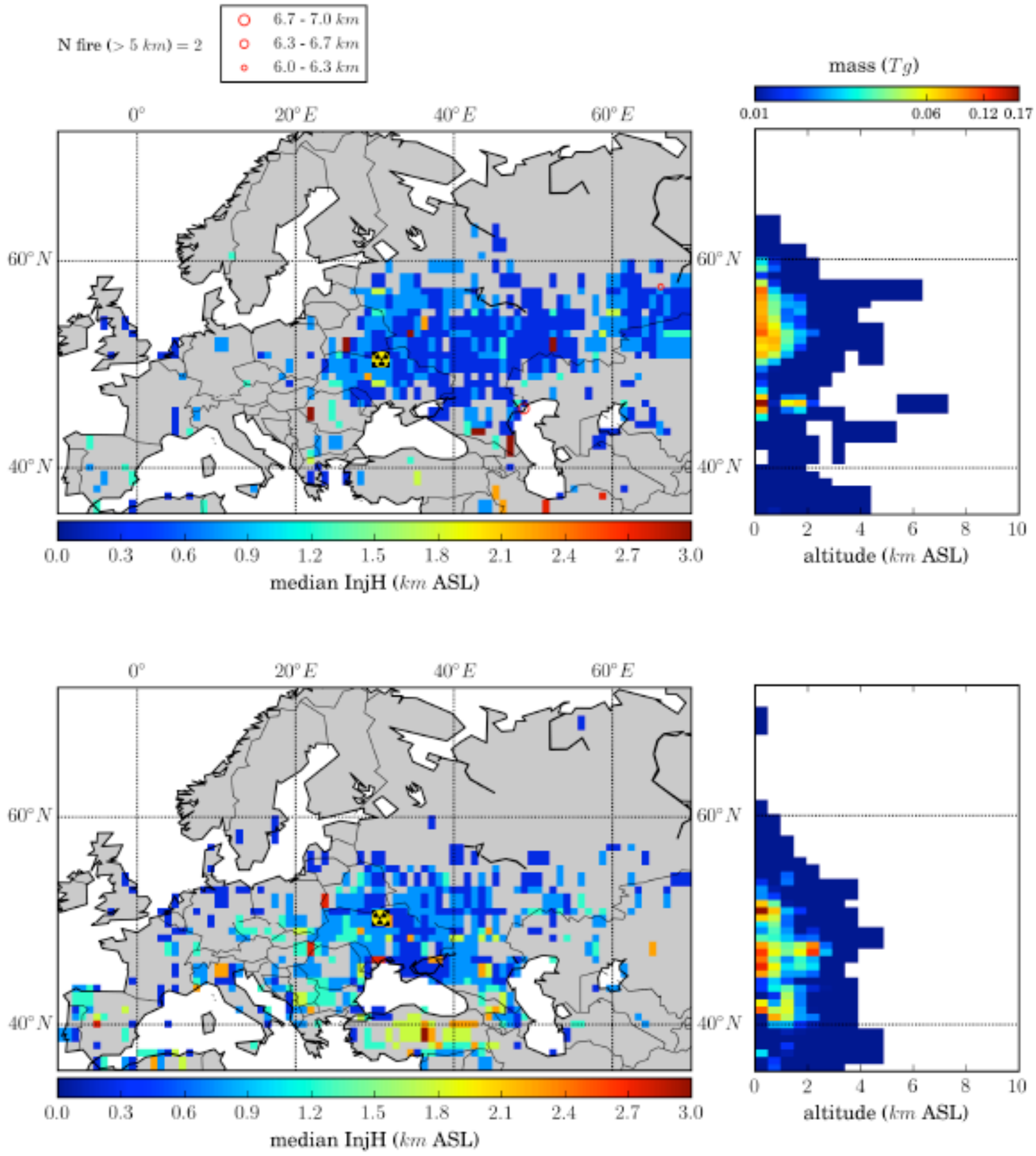

Fig. S 4. Median injection heights (km above sea level – ASL) and burned mass (Tg) of the fires occurring over Europe between 26 April and 2 May 2015 and between 9 and 14 August 2015 recorded by the PRMv2 [R. *The R Project for Statistical Computing version 3.2.3*. (2015) Available at: https://www.r-project.org (Accessed: 17th December 2015)].

TABLES FOR SUPPLEMENTS

Table S 1. Example of Landsat 8 OLI images used in remote sensing analysis for the spring and summer fires of 2015 in the CEZ.

Fire date	Image date	Path/row	State of the territory	ID image
April 27–29, 2015	April 23, 2013	182/024	Pre-fire	LC81820242013113LGN01
	April 26, 2014	182/024	Pre-fire	LC81820242014116LGN00
	April 29, 2015	182/024	Post-fire	LC81820242015119LGN00
	April 24, 2015	182/024	Post-fire	LC81810242015144LGN00
August 8–13, 2015	August 3, 2015	182/024	Pre-fire	LC81820242015215LGN00
	July 18, 2015	182/024	Pre-fire	LC81820242015199LGN00
	September 4, 2015	182/024	Post-fire	LC81820242015247LGN00
	September 20, 2015	181/024	Post-fire	LC81810242015263LGN00

Table S 2. Burn severity classifications according to the Landsat images used in the GIS analysis.

Burn severity	dNBR value	Type of fire	Type of fire
Unburned	–500 to 99	No fire	No fire
Low	100 – 269	Ground fire	Ground fire
Moderate-Low	270 – 439	Ground fire	
Moderate-High	440 – 659	Crown fire (if occupy more than 25% of stand area)	Crown fire (if occupy more than 25% of stand area)
High	660 – 1300	Crown fire (if occupy more than 25% of stand area)	
